# Combined low vitamin D and K status amplifies mortality risk: a prospective study

**DOI:** 10.1007/s00394-020-02352-8

**Published:** 2020-08-17

**Authors:** Adriana J. van Ballegooijen, Joline W. J. Beulens, Lyanne M. Kieneker, Martin H. de Borst, Ron T. Gansevoort, Ido P. Kema, Leon J. Schurgers, Marc G. Vervloet, Stephan J. L. Bakker

**Affiliations:** 1 Department of Nephrology, Amsterdam Cardiovascular Science, Amsterdam UMC, Location VUmc, Amsterdam, The Netherlands; 2 Department of Epidemiology & Biostatistics, Amsterdam Public Health Institute, Amsterdam UMC, Location VUmc, De Boelelaan 1117, Amsterdam, The Netherlands; 3 Department of Internal Medicine, University Medical Center Groningen, Groningen, The Netherlands; 4 Department of Laboratory Medicine, University Medical Center Groningen, Groningen, The Netherlands; 5 Department of Biochemistry, Maastricht University, Maastricht, The Netherlands

**Keywords:** Vitamin D, Vitamin K, All-cause mortality, Cardiovascular mortality

## Abstract

**Objective:**

To explore the association of both plasma vitamin D and K concentrations with all-cause mortality, cardiovascular mortality, and cardiovascular events in the general population.

**Methods:**

We studied 4742 participants of the Prevention of REnal and Vascular ENd-Stage Disease (PREVEND) Study. At baseline, vitamin D and K status was determined by measurement of 25-hydroxyvitamin D [25(OH)D] and dephosphorylated uncarboxylated matrix Gla protein (dp-ucMGP), respectively. Patients were categorized into: 25(OH)D < 50 or ≥ 50 nmol/L and dp-ucMGP < 361 or ≥ 361 pmol/L with 25(OH)D > 75 nmol/L and dp-ucMGP < 361 pmol/L as reference. Cause of death was coded according to International Classification of Diseases 9&10 codes from the 2001-2003 examination until date of death/event or censoring date (January 1st, 2017).

**Results:**

Mean age was 52.6 ± 11.9 years and 2513 (53%) were female. During a median of 14.2 year follow-up, 620 participants died of which 142 were due to cardiovascular causes. Combined low vitamin D and K status was present in 970 participants (20%) and was associated with a greater risk of all-cause mortality compared to high vitamin D and high vitamin K status group (*n* = 1424) after adjusting for potential confounders: hazard ratio 1.46 (95% confidence intervals 1.12–1.90). We observed similar trends, albeit non-significant for cardiovascular mortality, and cardiovascular events: 1.42 (0.79–2.55), 1.28 (0.93–1.77), respectively.

**Conclusions:**

Combined low vitamin D and K status are associated with increased all-cause mortality risk and possibly with cardiovascular mortality and cardiovascular events compared with adequate vitamin D and K status. Future studies should investigate the effect of combined vitamin D and K supplementation on clinical outcomes.

**Electronic supplementary material:**

The online version of this article (10.1007/s00394-020-02352-8) contains supplementary material, which is available to authorized users.

## Introduction

Nutritional deficiencies have been recognized as important contributors to disease and increased mortality. In this context, vitamin D gained much research interest. However, the potential role of other nutrient insufficiencies such as vitamin K received less attention. Vitamin K is required for more carboxylation (activation) of vitamin K-dependent proteins such as matrix Gla protein (MGP) and osteocalcin (bone Gla protein). Carboxylation of circulating matrix Gla protein reflects its capacity to inhibit calcification in the vasculature and is associated with coronary heart disease and mortality [1, 2]. Food sources that are high in vitamin K are: green leafy vegetables (broccoli, kale, spinach), fermented dairy products (quark, yoghurt, kefir, cheese), and egg yolks [3].

Vitamin D supplementation is mainly known for beneficial effects on bone density and fracture prevention [4]; however, this is currently under debate [5, 6]. Experimental studies suggest direct effects of vitamin D on vitamin K-dependent metabolism due to stimulation of the synthesis of vitamin K-dependent proteins by active vitamin D [7–10]. Excess vitamin D can induce a relative vitamin K deficiency. Vitamin K supplementation can overcome the untoward effect of excess vitamin D on calcification as demonstrated by lower calcium and phosphorus content in aorta and kidney [10]. The MGP-gene promoter contains a vitamin D response element, capable of a two-threefold enhanced MGP expression after vitamin D receptor activation [8]. The upregulation of MGP due to vitamin D would require vitamin K to ensure full activation of MGP for optimal functioning. This implies that the combination of both vitamins could provide enhanced protection against progressive vascular calcification.

The role of combined vitamin D and K status has only been investigated in studies on subclinical outcomes or in specific disease populations. In the general population, simultaneous low vitamin D and K status was cross-sectionally associated with arterial stiffness [11] and prospectively with increased blood pressure and incident hypertension [12]. Among kidney transplant recipients, low vitamin D and K status was associated with a twofold risk for mortality and graft failure [13] and particularly low vitamin K status was related to mortality in patients on vitamin D therapy. Worldwide, a large group of people use vitamin D supplements for fracture prevention, although new insights on the effects of vitamin D supplementation on other clinical endpoints have been disappointing [14]. These results could be explained by unnoticed interacting effects on MGP function. Despite these intriguing connections, no study has evaluated associations of vitamin D and K status with clinical outcomes in a general population on long-term outcomes.

Previous analyses of our cohort indicated that sufficient vitamin D or K alone are associated with survival benefits and reduced cardiovascular disease [15, 16]. We now extend these analyses by studying combined vitamin D and K status with the risk of all-cause mortality, cardiovascular mortality, and cardiovascular events. We also assessed vitamin K status among vitamin D supplement users and non-users.

## Methods

### Study population

The Prevention of REnal and Vascular ENd-Stage Disease (PREVEND) study is a prospective study on the development of albuminuria, renal, and cardiovascular disease. The study was initiated in 1997, when 40,856 inhabitants aged 28–75 years of the city Groningen, The Netherlands, were screened. Recruitment was based on urine albumin concentrations to enrich the cohort with individuals with albuminuria. All participants with a urinary albumin concentration of ≥ 10 mg/L *(n* = 7768) were invited, of whom 6000 participants were enrolled. In addition, a random sample of 3394 participants with urinary albumin concentrations < 10 mg/L were invited, of whom 2592 participants were enrolled. These 8592 individuals constituted the PREVEND cohort and completed an extensive examination between 1997 and 1998. Between 2001 and 2003, 6894 participants attended the first follow-up examination. In these participants, plasma samples were available for assessment of vitamin D and K status.

We excluded participants without measurement of 25-hydroxyvitamin D [25(OH)D] (*n* = 232), or dephosphorylated uncarboxylated matrix Gla protein (dp-ucMGP) (*n* = 1821), or vitamin K antagonist use (*n* = 99) leaving a final sample of 4742 participants. The study was approved by the medical ethics committee of the University Medical Center Groningen. All participants gave written informed consent and the study was in agreement with the Declaration of Helsinki.

### Measurement of vitamin D and K

Study personnel collected baseline blood samples in an 8–12 h fasted state. Samples were stored at − 80 °C until analysis in 2018. Plasma vitamin D concentrations were assessed by 25-hydroxyvitamin D_3_ concentrations [25(OH)D] using isotope dilution tandem mass spectrometry. Samples were extracted and analyzed by solid-phase extraction/liquid chromatography/tandem mass spectrometry using a Symbiosis online solid-phase extraction system (Spark Holland) [17]. The detection limit for 25(OH)D was 1.2 nmol/L, and intra-assay and inter-assay coefficients of variation were 6.7 and 7.2%, respectively.

To determine vitamin K status, plasma dp-ucMGP was measured at Coagulation Profile, Maastricht, The Netherlands, with a dual-antibody enzyme-linked immunoassay (InaKtif MGP/IDS-iSYS, Boldon, UK). In this assay, the capture antibody is directed against a non-phosphorylated matrix Gla protein epitope comprising amino acids 3-15 and the detection antibody against an uncarboxylated matrix Gla protein epitope that includes amino acids 35-49 [15]. The intra- and inter-assay coefficients of variation were 5.6% and 9.9%, respectively.

### All-cause and cardiovascular mortality

Mortality data were obtained from the municipal register, and the cause of death was obtained by linkage of the death certificates to the primary cause of death (physician-coded) from the Central Bureau of Statistics, The Netherlands. Cause of death was coded according to the Revision of the International Classification of Diseases (ICD-10). Cardiovascular death was classified with ICD-9 codes.

Data on non-fatal cardiovascular events were derived from hospital discharge diagnoses obtained from the Dutch National Registry PRISMANT [18]. This comprised cardiac, cerebrovascular, and peripheral events. These non-fatal events were combined with cardiovascular mortality into cardiovascular events. We defined the time at risk as the elapsed time from the 2001–2003 examination until date of death/event or censoring date (January 1st, 2017).

### Other baseline variables

Participants completed questionnaires to determine detailed information on demographic, health-related behaviors, diagnosis of cardiovascular and renal disease, family history, ethnicity, attained education, medication use, and frequency of sports [19]. Smoking status was categorized as never/former/current. Information on medication use was combined with information of prescribed medication. Body mass index (BMI) was based on measured height and weight (kg/m^2^). Prior non-fatal cardiovascular disease (CVD) was classified as self-report for coronary heart disease and stroke or based on hospital records.

Serum creatinine, total cholesterol, high-density lipoprotein cholesterol, triglycerides, C-reactive protein, and glucose were determined as previously described [19]. Serum cystatin C concentrations were measured by Gentian Cystatin C Immunoassay (Gentian AS, Moss, Norway) on a Modular analyzer (Roche Diagnostics Mannheim, Germany) and circulating calcium and phosphate were determined in lithium heparin plasma. Estimated glomerular filtration rate (eGFR) was calculated based on combined creatinine and cystatin C equation [20].

### Statistical analysis

We categorized 25(OH)D as < 50/≥ 50 nmol/L, the clinical cut-off value for vitamin D insufficiency. For vitamin K status, no clinical cut-off value is currently available and we categorized dp-ucMGP by the median 361 pmol/L and consider this as low vs high vitamin K status. We defined four groups of vitamin D and K status with the 25(OH)D category above 50 mmol/L and dp-ucMGP < 361 pmol/L (indicating sufficient MGP carboxylation by vitamin K) as reference. We also investigated vitamin K status among vitamin D supplement users and non-users. In addition, we categorized dp-ucMGP based on 500 pmol/L, since some evidence suggests this cut-off for functional vitamin K insufficiency [15].

We summarized continuous values by mean and standard deviation or median and interquartile range (IQR) for the vitamin D and K groups. To visualize the relationship between 25(OH)D and dp-ucMGP concentrations, we constructed a locally weighted regression plot.

Differences in survival between the vitamin D and K groups were graphically displayed using Kaplan–Meier curves followed by log-rank. We used Cox regression analysis to estimate hazard ratios and 95% confidence intervals of the vitamin D and K groups with mortality outcomes adjusted for characteristics that could plausibly confound this association. Our first model examined associations adjusted for age, sex, and a cosinor model to account for the season of blood sampling [21]. In our second model, we added smoking, education, frequency of sports, BMI, and systolic blood pressure and glucose. In the final model, we added eGFR, blood pressure lowering, and cholesterol lowering medication use and prior CVD. Due to fewer events for cardiovascular mortality, the association was only adjusted for model 2.

For more specific analysis, we combined four clinical cut-off values of 25(OH)D < 25/25-50/50-75/> 75 nmol/L and dp-ucMGP < 361/≥ 361 pmol/L into eight categories of vitamin status with the category 25(OH)D > 75 and dp-ucMGP < 361 as reference. We tested for interaction by comparing the likelihoods of the nested models with and without the specific interaction terms for vitamin D and K status categories with all-cause mortality using the likelihood ratio test. This analysis with eight categories was only performed for all-cause mortality, since we did not have enough statistical power for cardiovascular mortality.

We examined additive interaction, since the product term reflects multiplicative interaction. It has been argued that interaction estimated as additive interaction better reflects biological interaction and the synergy index is the measure of choice [22]. Synergy index (ratio combined effect and individual effects): S = 1 no interaction/additivity; S > 1 means positive interaction/additivity; S < 1 negative interaction/no additivity; S can range from 0 to infinity:

$$ {\text{Synergy index}} = \frac{{RR^{ + + } - 1}}{{\left( {\left( {RR^{ + - } - 1} \right) + \left( {RR^{ - + } - 1} \right)} \right)}}. $$

To evaluate continuous associations, we constructed cubic smoothing splines between dp-ucMGP and mortality risk stratified by 25(OH)D ≤ 50/> 50 nmol/L. To test the robustness of the associations, we excluded participants on vitamin D supplements and those with prior CVD.

We conducted analyses using SPSS, version 26 (SPSS Inc., Chicago, IL, USA) and R-version 3.61 and considered a two-sided p < 0.05 to be statistically significant.

### Patient and public involvement

This research was performed without patient involvement in study design, interpretation of results, and writing of this document.

## Results

### Study population

Among the 4742 PREVEND participants, mean age was 52.6 ± 11.9 years and 2513 (53%) were female. Approximately 27% of the participants were current smokers, 25% never used alcohol, 16% used blood pressure lowering medication, and 8% had a prior CVD (*n* = 381).

Excluded participants (*n* = 2152) were older, more likely to be men, had higher systolic blood pressure, higher BMI, lower eGFR, and more likely to use blood pressure lowering treatment.

### Description of 25(OH)D and dp-ucMGP

**Fig. 1:**
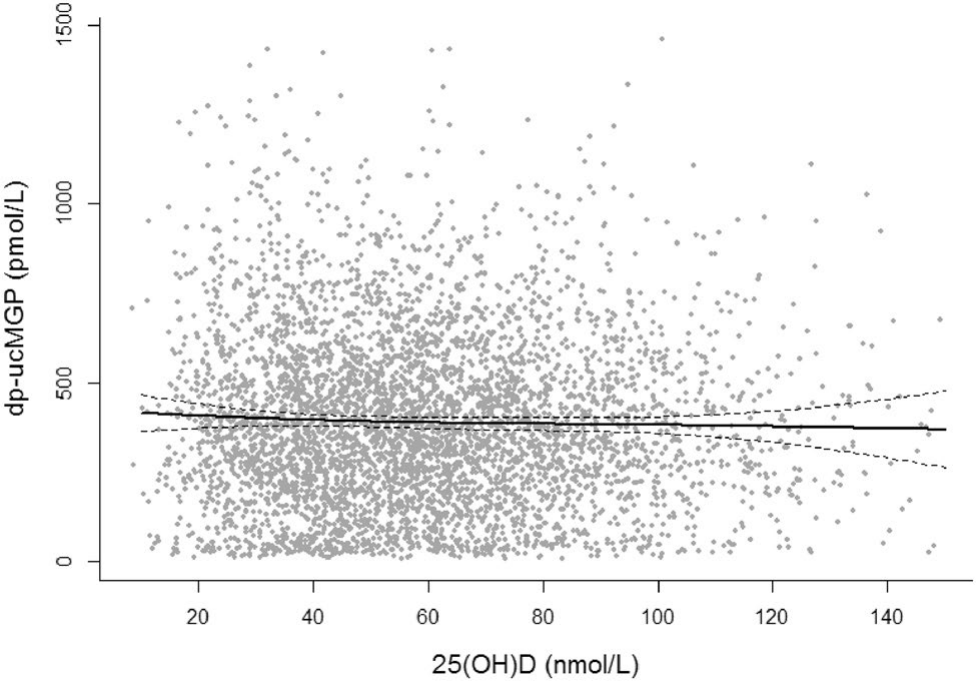
Lowess of plasma 25(OH)D and dp-ucMGP concentrations with 95% confidence intervals among 4742 PREVEND participants

Both plasma 25(OH)D and dp-ucMGP were approximately normally distributed with mean concentrations of 59.4 ± 26.0 nmol/L and 394 ± 260 pmol/L, respectively. Plasma 25(OH)D was not associated with dp-ucMGP (Fig. 1).

Vitamin D (25(OH)D < 25 nmol/L) was found among 6% and vitamin D (25(OH)D < 50 nmol/L) was found among 34% of participants. Vitamin K insufficiency, based on ≥ 500 pmol/L, was present among 29%. Combined low vitamin D and K status (median dp-ucMGP 361 pmol/L) was present in 970 participants (20%) and was associated with younger age, higher BMI, triglyceride, CRP, glucose and PTH values, and lower eGFR and more medication prescriptions (Table 1).

**Table 1:** Baseline characteristics according to categories of 25-hydroxyvitamin D and dephosphorylated uncarboxylated matrix gla protein in 4,742 PREVEND participants

	Categories of 25(OH)D (nmol/L) and dp-ucMGP status (pmol/L)			
	25(OH)D < 50 dp-ucMGP ≥ 361	25(OH)D ≥ 50 dp-ucMGP ≥ 361	25(OH)D < 50 dp-ucMGP < 361	25(OH)D ≥ 50 dp-ucMGP < 361
Demographics				
*N*	970	1406	942	1424
Women	507 (52%)	682 (49%)	515 (55%)	809 (57%)
Age (year)	56.8 ± 12.6	55.7 ± 11.7	50.2 ± 11.2	48.2 ± 10.2
BMI (kg/m^2^)	28.1 ± 4.8	26.9 ± 3.9	26.1 ± 4.2	25.1 ± 4.2
Education				
Low	368 (38%)	464 (33%)	260 (28%)	314 (22%)
Medium	352 (36%)	524 (37%)	370 (39%)	544 (38%)
High	250 (26%)	415 (30%)	312 (33%)	566 (40%)
Smoking status				
Never	308 (32%)	407 (29%)	304 (32%)	419 (30%)
Former	421 (43%)	673 (48%)	320 (34%)	560 (40%)
Current	232 (24%)	309 (23%)	316 (34%)	430 (30%)
Frequency of sports				
None	673 (70%)	826 (59%)	620 (66%)	659 (47%)
Once per week	172 (18%)	317 (23%)	200 (21%)	425 (30%)
At least twice per week	113 (12%)	245 18%)	119 (13%)	321 (23%)
Prior CVD	107 (11%)	159 (11%)	61 (6%)	54 (4%)
SBP (mm Hg)	131 ± 20	128 ± 19	122 ± 17	120 ± 15
DBP (mm Hg)	75 ± 9	74 ± 9	72 ± 9	71 ± 9
Alcohol use (drinks)				
None	332 (34%)	305 (22%)	276 (29%)	254 (18%)
Light (1–4 p/month)	178 (20%)	207 (15%)	172 (18%)	243 (17%)
Moderate (2–7 p/week)	224 (23%)	462 (33%)	301 (32%)	533 (38%
Heavy (1–3/day)	167 (17%)	350 (25%)	158 (17%)	328 (23%)
Very heavy (≥ 4/day)	61 (6%)	66 (5%)	33 (4%)	49 (4%)
Metabolic				
Total cholesterol (mmol/L)	5.5 ± 1.1	5.5 ± 1.0	5.4 ± 1.1	5.3 ± 1.0
Triglycerides (mmol/L)	1.4 ± 1.0	1.3 ± 0.9	1.3 ± 1.0	1.2 ± 0.8
Glucose (mmol/L)	5.1 ± 1.0	4.9 ± 0.8	4.9 ± 1.0	4.7 ± 0.6
C-reactive protein (mg/L)	1.6 [0.8-3.3]	1.4 [0.7-2.8]	1.1 [0.5-2.7]	1.0 [0.5-2.7]
eGFR (mL/min/1.73 m^2^)	87 ± 18	88 ± 16	97 ± 15	98 ± 14
25(OH) D (nmol/L)	35 ± 9	75 ± 20	36 ± 9.2	76 ± 20
Dp-ucMGP (pmol/L)	603 ± 242	573 ± 214	194 ± 103	208 ± 102
PTH (pmol/L)	5.9 ± 2.4	5.0 ± 1.4	5.3 ± 1.5	4.6 ± 1.2
Medication use				
Vitamin D supplement	6 (1%)	12 (1%)	5 (1%)	5 (1%)
Lipid lowering	111 (12%)	152 (11%)	68 (7%)	77 (6%)
Blood pressure lowering	216 (23%)	279 (20%)	114 (12%)	140 (10%)
Glucose lowering	22 (2%)	15 (1%)	6 (1%)	1 (0%)

Values are presented as means with standard deviations, median with interquartile range, or percentages

*25(OH)D* 25-hydroxyvitamin D, *dp*-*ucMGP* dephosphorylated uncarboxylated matrix gla protein, *BMI* body mass index, *CVD* cardiovascular disease, *SPB* systolic blood pressure, *DBP* diastolic blood pressure, *HDL* high-density lipoprotein, *eGFR* estimated glomerular filtration rate, *PTH* parathyroid hormone

Among the 28 vitamin D supplement users, median dp-ucMGP concentrations were higher 479 (IQR 116–697) compared to no vitamin D supplement use dp-ucMGP: 362 (IQR 218-528) pmol/L (*N* = 4496).

### Mortality

During a median follow-up of 14.2 years (IQR 11.2–14.8), 620 participants (13%) died, of which 142 were due to cardiovascular causes (23%). In total, 466 participants had a cardiovascular event (fatal and non-fatal), of which 157 (34%) had a prior history of CVD.

**Fig. 2:**
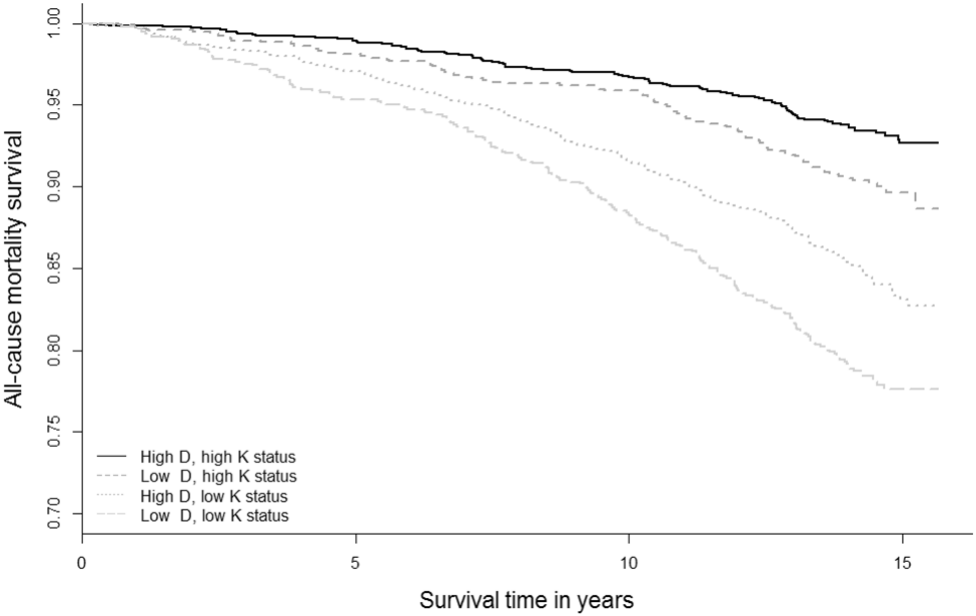
Survival curves for 25-hydroxyvitamin D and dephosphorylated uncarboxylated matrix gla protein categories with all-cause mortality in 4742 PREVEND participants. All-cause mortality: *N *= 620 died, log-rank *P *< 0.001. Vitamin D status: 25(OH)D < 50/≥ 50 nmol/L, vitamin K status dp-ucMGP < 361/≥ 361 pmol/L

Survival curves for combined vitamin D and K categories differed for all-cause mortality and cardiovascular mortality, log rank *p* < 0.001 (Fig. 2). Combined low vitamin D and K status was associated with a higher risk of all-cause mortality after follow-up with an incidence of 22% vs 7% for the combined high vitamin D and K status group (Table 2): HR 1.44 (1.11–1.87), P-interaction < 0.001. After adjusting for potential confounders, the risk estimate became slightly stronger: 1.46 (1.12–1.90). The risks for only low vitamin D or low vitamin K status were: 1.13 (0.87–1.46) and 1.09 (0.81–1.48), respectively. The synergy index was 2.09.

**Table 2:** Associations of combined 25-hydroxyvitamin D and dephosphorylated uncarboxylated matrix gla protein categories with all-cause mortality and cardiovascular mortality in 4742 PREVEND participants

	Categories of 25(OH)D nmol/L and dp-ucMGP status (pmol/L)			
	25(OH)D < 50 dp-ucMGP ≥ 361	25(OH)D ≥ 50 dp-ucMGP ≥ 361	25(OH)D < 50 dp-ucMGP < 361	25(OH)D ≥ 50 dp-ucMGP < 361
*N*	970	1406	942	1424
All-cause mortality				
No. of cases	209 (22%)	220 (16%)	95 (10%)	96 (7%)
Person-years	12,163	17,492	11,946	18,051
Incidence rate/1000 p/y	17.2	12.6	8.0	5.3
Model 1	1.44 (1.11-1.87)	1.13 (0.87-1.45)	1.20 (0.89-1.62)	1.00 (Ref)
Model 2	1.45 (1.11-1.89)	1.18 (0.91-1.53)	1.07 (0.79-1.45)	1.00 (Ref)
Model 3	1.46 (1.12-1.90)	1.13 (0.87-1.46)	1.09 (0.81-1.48)	1.00 (Ref)
Cardiovascular mortality				
No. of cases	52 (5%)	52 (4%)	21 (2%)	17 (1%)
Person-years, y	12,920	19,162	13,235	20,200
Incidence rate/1000 p/y	4.0	2.7	1.6	0.8
Model 1	1.54 (0.87-2.74)	1.17 (0.67-2.09)	1.42 (0.74-2.73)	1.00 (Ref)
Model 2	1.42 (0.79-2.55)	1.23 (0.69-2.18)	1.25 (0.65-2.43)	1.00 (Ref)
Cardiovascular events				
No. of cases	142 (15%)	182 (13%)	73 (8%)	69 (5%)
Person-years, y	11,624	16,632	11,604	17,649
Incidence rate/1000 p/y	12.2	10.9	6.3	3.9
Model 1	1.60 (1.19-2.15)	1.46 (1.10-1.93)	1.39 (1.00-1.93)	1.00 (Ref)
Model 2	1.41 (1.03-1.91)	1.38 (1.03-1.85)	1.30 (0.93-1.82)	1.00 (Ref)
Model 3	1.28 (0.93-1.77)	1.15 (0.85-1.56)	1.27 (0.90-1.78)	1.00 (Ref)

Hazard ratios and 95% confidence intervals derived from Cox proportional hazard models

*25(OH)D* 25-hydroxyvitamin D, *dp*-*ucMGP* dephosphorylated uncarboxylated matrix gla protein. To convert 25(OH)D to ng/mL divide by 2.5

25(OH)D ≥ 50: sufficient vitamin D status, dp-ucMGP ≥ 361 pmol/L: vitamin K deficiency

Model 1 adjusted for age, sex, and a cosinor model to account for time of the year

Model 2 includes model 1 plus smoking (never/former/current), body mass index (kg/m^2^), education (3 categories), frequency of sports (3 categories), systolic blood pressure (mm Hg), and glucose (mmol/L)

Model 3 includes model 2 and estimated glomerular filtration rate (mL/min/1.73 m^2^), blood pressure lowering and cholesterol lowering medication use, and prior cardiovascular disease

For cardiovascular mortality, we observed trends in the same direction, although the risk estimates were not statistically significant. The low vitamin D and K status group was associated with a 42% greater risk for cardiovascular mortality: 1.42 (0.79–2.55) (model 2). For cardiovascular events, also similar trends were observed. In model 2, the risk estimate for the low vitamin D and K group was: 1.41 (1.03–1.91); however, the relationship attenuated after adjusting for cardiovascular risk factors: 1.28 (0.93–1.77).

**Fig. 3:**
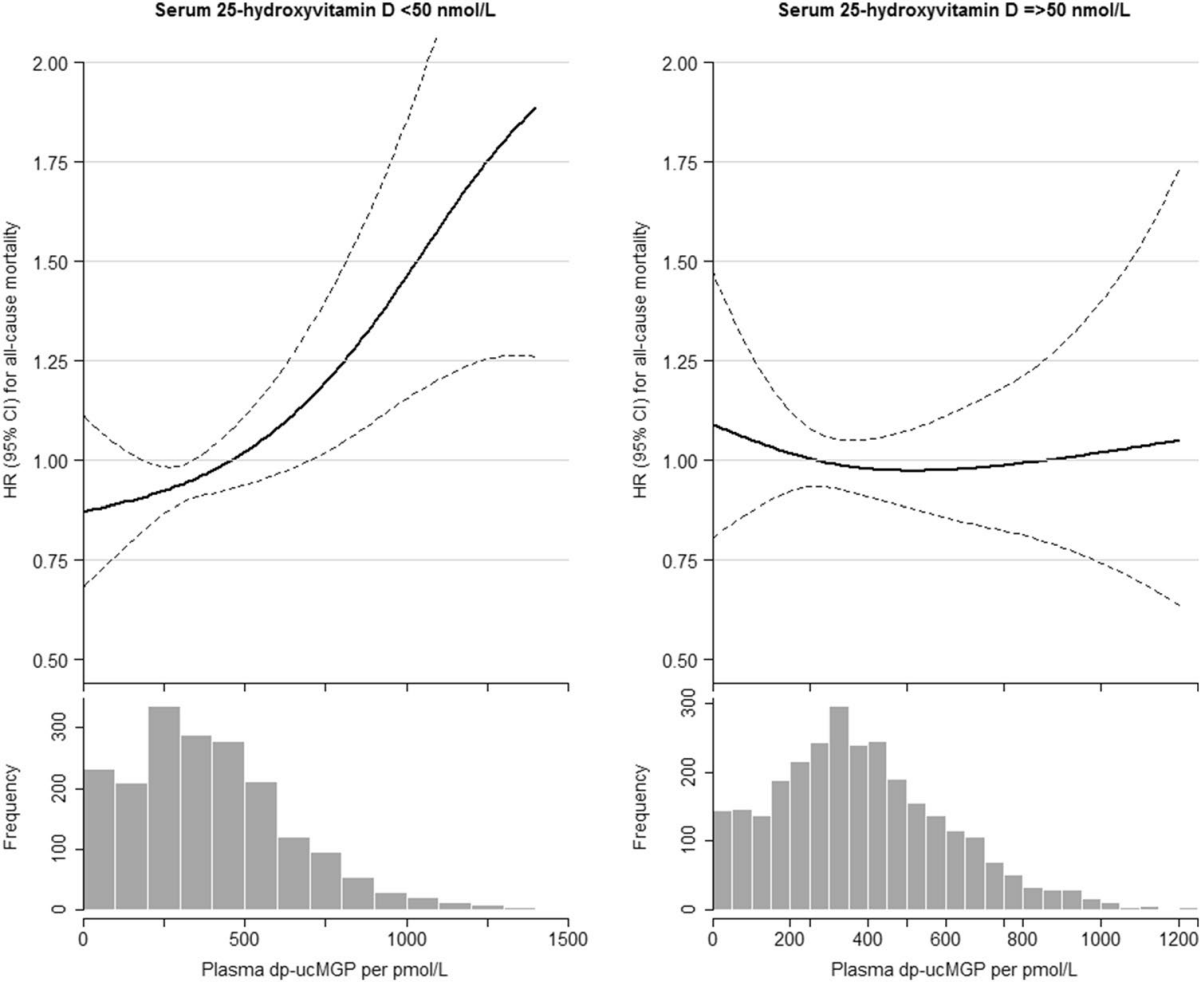
Continuous associations for dp-ucMGP with all-cause mortality stratified by vitamin D clinical cut-off categories. Dp-ucMGP: dephosphorylated uncarboxylated matrix gla protein. Adjusted for age and sex. 25(OH)D < 50 nmol/L: *N *= 1912; 25(OH)D ≥ 50 nmol/L: *N *= 2830

Based on eight categories 25(OH)D dp-ucMGP, the risk estimates were more pronounced (P-interaction = 0.010) (Table 3). The lowest vitamin D group (25(OH)D < 25 nmol/L) combined with low vitamin K (dp-ucMGP ≥ 361) showed the greatest mortality risk: 2.27 (1.40–3.69). All risk estimates for low vitamin K status were higher compared with high vitamin K status within the same vitamin D category. For low vitamin D status both < 25 nmol/L and 25–50 nmol/L, the risk estimates for low vitamin K status were stronger associated with mortality risk than high vitamin K status. This is also depicted by the continuous association of dp-ucMGP and mortality risk stratified by low/high vitamin D status (Fig. 3). Only for low vitamin D status, a steep positive association was present for dp-ucMGP concentrations and mortality risk.

**Table 3:** Associations of median dephosphorylated uncarboxylated matrix gla protein with all-cause mortality by clinical 25-hydroxyvitamin D cut-off values in 4742 PREVEND participants

	Plasma 25-hydroxyvitamin D/L categories (nmol/L)				*P*-for interaction
	< 25	≥ 25–50	≥ 50–75	≥ 75	
	*N *= 302	*N *= 1610	*N *= 1637	*N *= 1193	
Dp-ucMGP pmol/L	431 ± 299	393 ± 257	389 ± 230	385 ± 233	
All-cause mortality					
No of cases	21 (16%)	74 (9%)	58 (7%)	38 (6%)	
dp-ucMGP < 361 pmol/L	1.79 (0.98–3.26)	1.16 (0.76–1.76)	1.21 (0.78–1.87)	Ref 1.0	0.010
No of cases	40 (24%)	169 (21%)	137 (17%)	83 (14%)	
dp-ucMGP ≥ 361 pmol/L	2.27 (1.40–3.69)	1.54 (1.04–2.28)	1.25 (0.84–1.84)	1.30 (0.85–1.97)	

Hazard ratios and 95% confidence intervals adjusted for: age, sex, and a cosinor model to account for time of the year, smoking (never/former/current), body mass index (kg/m^2^), education (three categories), frequency of sports (three categories), systolic blood pressure (mm Hg), estimated glomerular filtration rate (mL/min/1.73 m^2^), glucose (mmol/L), blood pressure lowering and cholesterol lowering medication use, and prior cardiovascular disease

*dp*-*ucMGP* dephosphorylated uncarboxylated matrix gla protein

Sensitivity analyses excluding vitamin D supplement users (*n* = 28) and participants with prior CVD (*n* = 381) revealed similar results for all-cause and cardiovascular mortality, but were less pronounced for cardiovascular events (supplemental Tables 1, 2). When dp-ucMGP was categorized based on dp-ucMGP 500 pmol/L, the results attenuated, but remained statistically significant (Supplemental Table 3). The results were also similar when 25(OH)D was divided by < 75 vs ≥ 75 nmol/L meaning that there is no additional benefit of higher 25(OH)D concentrations on all-cause mortality risk (Supplemental Table 4).

## Discussion

In a cohort of middle-aged individuals from the general population, combined low vitamin D and K status was present in 20% and was associated with a greater risk of all-cause mortality over a median follow-up of 14.2 years compared to adequate vitamin D and K status. The combined association of low vitamin D and K status with mortality was greater than the sum of low vitamin D and K status alone, indicating that the combined insufficiency amplifies mortality risk. For cardiovascular mortality and cardiovascular events, we observed similar trends although not statistically significant.

To our knowledge, this is the first study that investigated the association of combined vitamin D and K status with mortality outcomes in the general population. The previous studies have investigated incident hypertension and arterial stiffness in general populations [11, 12] or were conducted in a specific patient group [13]. Among kidney transplant recipients, the combination of low vitamin D and K status was highly prevalent and was associated with a 150% increased risk of all-cause mortality compared to high vitamin D and K status after long-term follow-up [13]. These studies are in line with our results in the general population.

Other studies that investigated vitamin D and vitamin K status found that the combination of low concentrations was associated with decline in physical performance [23] or with hip fractures in older adults [24, 25]. These two studies measured vitamin K status as plasma vitamin K_1_ concentrations, which is a global indicator of vitamin K intake and mainly reflects intake of previous days, because of its half-life time 1-3 h [26], and, therefore, may not appropriately reflect functional vitamin K status. None of these studies had information on vitamin D supplement use to perform stratified analyses.

These results for unfavorable measures of cardiovascular health, worse physical performance, fractures, and increased mortality could all be related to calcium crystal deposition in the vasculature and the kidney. With low amounts of vitamin D and K, the processes to activate vitamin K-dependent proteins to stimulate bone mineralization and inhibit soft-tissue calcification are diminished, which could result in vascular calcification and death. This process could possibly be counteracted by combined vitamin D and K supplementation as beneficial effects on bone mineral density and elastic properties of the arterial vessel wall have been reported compared to vitamin D supplementation alone [27, 28]. This is also in line with randomized trials that compared a vitamin D supplement vs a vitamin D + K supplement and observed synergistic effects for the combination of vitamin D and K [29, 30]. However, we cannot exclude that vitamin K status is not associated with mortality in the context of sufficient vitamin D status.

In our study with baseline measures between 2001 and 2003, only 28 participants used vitamin D supplements. Among the vitamin D supplement users, median dp-ucMGP concentrations were higher compared to non-users (479 vs 362 pmol/L). Due to the small subset of supplement users, it is hard to draw conclusions about vitamin D supplementation. We expect that increased concentration of 25(OH)D are different from vitamin D supplements compared to increased 25(OH)D concentrations due to increased UVB-exposure, which is usually due to an active outdoor lifestyle [31]. For this reason, we restricted the analysis to non-vitamin D supplement users. Similar observations were found among kidney transplant recipients with higher dp-ucMGP concentrations among people on vitamin D therapy 1281 vs 974 pmol/L among non-users [13]. Also in a randomized-controlled trial, vitamin D supplementation for 6 months increased inactive dp-ucMGP compared to placebo in older adults without vitamin K antagonist or multi-vitamin use [32]. The use of vitamin D supplements has increased tremendously over the last decade and, therefore, more research is needed to investigate whether monotherapy of vitamin D can worsen vitamin K status in various populations.

### Strengths and limitations

Our study has several strengths. First, this study is a large cohort of 4742 participants with both 25(OH)D and dp-ucMGP measured using state-of-the art methods. The individuals within PREVEND had a low prevalence of CVD, which allowed us to study associations between vitamin D and K status in the general population with minimal comorbidities.

For plasma dp-ucMGP, no clinical cut-off value has been developed although the results attenuated only slightly with the cut-off of 500 and were still statistically significant meaning that results were robust [15]. The results of our study were similar after excluding vitamin D supplement users or participants with prior CVD at baseline for all-cause and cardiovascular mortality, except for cardiovascular events. A large part of the participants with a cardiovascular event experienced a prior event (34%), meaning that the relationship was driven by their higher baseline risk, since the results attenuated when participants with prior CVD were excluded.

Our study has also some limitations. Vitamin D and K status were only measured at baseline. Despite this fact, we showed that the simultaneous low concentrations of vitamin D and K are associated with all-cause mortality independent of classic risk factors. For more specific analyses with eight categories of 25(OH)D and dp-ucMGP, we did not have sufficient statistical power for cardiovascular mortality. Given the prolonged decrease in cardiovascular death in many countries worldwide, future studies should be sufficiently large to study cardiovascular mortality [33]. Future studies could benefit from a meta-analysis of prospective individual patient data to have sufficient statistical power for cardiovascular mortality as has been done for serum vitamin and mortality [34].

Moreover, our study was observational in nature and, therefore, the possibility of residual confounding cannot be excluded. Sufficient vitamin D and K status are related to a healthy lifestyle. However, we adjusted for smoking, BMI, and sports making it less likely that a healthy lifestyle explains our findings, although we cannot exclude residual confounding by for example a healthy diet. Also, we did not have information on fracture incidence, which is likely to benefit from sufficient vitamin status. The samples have been stored at− 80 °C after collection and no data are available whether potential sample degradation could have affected our results. However, sample degradation would be systematic for all samples and would most likely dilute the observed associations.

In conclusion, combined low vitamin D and K status was present in 20% of the participants and was associated with a greater risk of mortality after 14.2 year follow-up. The association of combined low vitamin D and K status with mortality was greater than one vitamin insufficiency alone, and amplified these risks. Future studies should compare whether the effect of combined vitamin D and K supplementation may lead to improved cardiovascular outcomes.

## Electronic supplementary material

Below is the link to the electronic supplementary material.

Supplementary material 1 (DOCX 19 kb)
