# Supplementary material for: Combined low vitamin D and K status amplifies mortality risk: a prospective study
Source: Eur J Nutr. 2020 Aug 17;60(3):1645–54. doi: 10.1007/s00394-020-02352-8 (PMC7987611; doi:10.1007/s00394-020-02352-8)
Supplement: Supplementary file 1 — Supplementary material 1 (DOCX 19 kb) [file 394_2020_2352_MOESM1_ESM.docx]

**Supplemental table 1**: Excluding vitamin D supplement users

Associations of combined 25-hydroxyvitamin D and desphosphorylated uncarboxylated matrix gla protein categories with all-cause mortality and cardiovascular mortality in 4,496 PREVEND participants

|  | Categories of 25(OH)D nmol/L and dp-ucMGP status (pmol/L) | | | |
| --- | --- | --- | --- | --- |
|  | 25(OH)D <50  dp-ucMGP ≥361 | 25(OH)D ≥50  dp-ucMGP ≥361 | 25(OH)D <50  dp-ucMGP <361 | 25(OH)D ≥50  dp-ucMGP <361 |
| N | 944 | 1,365 | 914 | 1373 |
| **All-cause mortality** |  |  |  |  |
| No. of cases | 203 (22%) | 217 (16%) | 90 (10%) | 95 (7%) |
| Model 1 | 1.38 (1.08-1.78) | 1.11 (0.87-1.41) | 1.18 (0.88-1.58) | 1.00 (Ref) |
| Model 2 | 1.39 (1.07-1.80) | 1.16 (0.90-1.50) | 1.10 (0.82-1.48) | 1.00 (Ref) |
| Model 3 | 1.43 (1.10-1.87) | 1.12 (0.86-1.45) | 1.06 (0.78-1.44) | 1.00 (Ref) |
| **Cardiovascular mortality** |  |  |  |  |
| No. of cases | 51 (5%) | 51 (4%) | 19 (2%) | 17 (1%) |
| Model 1 | 1.82 (0.89-3.72) | 1.46 (0.72-2.98) | 1.18 (0.81-2.73) | 1.00 (Ref) |
| Model 2 | 1.48 (0.71-3.09) | 1.30 (0.85-2.70) | 1.09 (0.47-2.53) | 1.00 (Ref) |
| **Cardiovascular events** |  |  |  |  |
| No. of cases | 141 (15%) | 177 (13%) | 70 (8%) | 68 (5%) |
| Model 1 | 1.60 (1.19-2.15) | 1.43 (1.07-1.90) | 1.37 (0.98-1.91) | 1.00 (Ref) |
| Model 2 | 1.41 (1.04-1.92) | 1.36 (1.02-1.83) | 1.28 (0.91-1.80) | 1.00 (Ref) |
| Model 3 | 1.29 (0.93-1.78) | 1.14 (0.84-1.55) | 1.25 (0.88-1.76) | 1.00 (Ref) |

Hazard ratios and 95% confidence intervals derived from Cox proportional hazard models.

Abbreviations: 25(OH)D: 25-hydroxyvitamin D, dp-ucMGP: dephosphorolated uncarboxylated matrix gla protein. To convert 25(OH)D to ng/mL divide by 2.5.

25(OH)D ≥ 50: sufficient vitamin D status, dp-ucMGP ≥361 pmol/L: vitamin K deficiency.

Model 1 adjusted for age, sex, and a cosinor model to account for time of the year

Model 2 includes model 1 plus smoking (never/former/current), body mass index (kg/m^2^), education (3 categories), frequency of sports (3 categories), systolic blood pressure (mm Hg), glucose (mmol/L)

Model 3 includes model 2 and estimated glomerular filtration rate (mL/min/1.73m^2^), blood pressure and cholesterol lowering medication use

**Supplemental table 2:** Excluding prior CVD

Associations of combined 25-hydroxyvitamin D and desphosphorylated uncarboxylated matrix gla protein categories with all-cause mortality and cardiovascular mortality in 4,475 PREVEND participants

|  | Categories of 25(OH)D nmol/L and dp-ucMGP status (pmol/L) | | | |
| --- | --- | --- | --- | --- |
|  | 25(OH)D <50  dp-ucMGP ≥361 | 25(OH)D ≥50  dp-ucMGP ≥361 | 25(OH)D <50  dp-ucMGP <361 | 25(OH)D ≥50  dp-ucMGP <361 |
| N | 894 | 1292 | 900 | 1,389 |
| **All-cause mortality** |  |  |  |  |
| No. of cases | 172 (19%) | 166 (13%) | 78 (9%) | 84 (6%) |
| Model 1 | 1.43 (1.09-1.87) | 1.04 (0.80-1.36) | 1.15 (0.84-1.56) | 1.00 (Ref) |
| Model 2 | 1.38 (1.04-1.82) | 1.11 (0.84-1.46) | 1.07 (0.78-1.48) | 1.00 (Ref) |
| Model 3 | 1.39 (1.04-1.86) | 1.05 (0.79-1.39) | 0.99 (0.71-1.38) | 1.00 (Ref) |
| **Cardiovascular mortality** |  |  |  |  |
| No. of cases | 38 (4%) | 39 (3%) | 14 (2%) | 11 (1%) |
| Model 1 | 1.74 (0.88-3.47) | 1.49 (0.75-2.94) | 1.30 (0.59-2.89) | 1.00 (Ref) |
| Model 2 | 1.47 (0.70-3.05) | 1.57 (0.77-3.21) | 1.17 (0.52-2.67) | 1.00 (Ref) |
| **Cardiovascular events** |  |  |  |  |
| No. of cases | 91 (10%) | 119 (9%) | 46 (5%) | 53 (4%) |
| Model 1 | 1.40 (0.99-1.98) | 1.36 (0.98-1.89) | 1.16 (0.78-1.72) | 1.00 (Ref) |
| Model 2 | 1.12 (0.78-1.61) | 1.25 (0.89-1.76) | 1.03 (0.68-1.53) | 1.00 (Ref) |
| Model 3 | 1.05 (0.72-1.54) | 1.19 (0.84-1.70) | 1.00 (0.66-1.51) | 1.00 (Ref) |

Hazard ratios and 95% confidence intervals derived from Cox proportional hazard models.

Abbreviations: 25(OH)D: 25-hydroxyvitamin D, dp-ucMGP: dephosphorolated uncarboxylated matrix gla protein. To convert 25(OH)D to ng/mL divide by 2.5.

25(OH)D ≥ 50: sufficient vitamin D status, dp-ucMGP ≥361 pmol/L: vitamin K deficiency.

Model 1 adjusted for age, sex, and a cosinor model to account for time of the year

Model 2 includes model 1 plus smoking (never/former/current), body mass index (kg/m^2^), education (3 categories), frequency of sports (3 categories), systolic blood pressure (mm Hg), glucose (mmol/L)

Model 3 includes model 2 and estimated glomerular filtration rate (mL/min/1.73m^2^), blood pressure lowering and cholesterol lowering medication use, prior cardiovascular disease

**Supplemental table 3.** Dp-ucMGP </≥500 pmol/L

Associations of combined 25-hydroxyvitamin D and desphosphorylated uncarboxylated matrix gla protein categories with all-cause mortality and cardiovascular mortality in 4,742 PREVEND participants

|  | Categories of 25(OH)D nmol/L and dp-ucMGP status (pmol/L) | | | |
| --- | --- | --- | --- | --- |
|  | 25(OH)D <50  dp-ucMGP ≥500 | 25(OH)D ≥50  dp-ucMGP ≥500 | 25(OH)D <50  dp-ucMGP <500 | 25(OH)D ≥50  dp-ucMGP <500 |
| N | 569 | 784 | 1,343 | 2,046 |
| **All-cause mortality** |  |  |  |  |
| No. of cases | 154 (27%) | 138 (18%) | 150 (11%) | 178 (9%) |
| Model 1 | 1.35 (1.08-1.68) | 0.98 (0.78-1.23) | 1.16 (0.93-1.45) | 1.00 (Ref) |
| Model 2 | 1.37 (1.09-1.72) | 1.04 (0.82-1.30) | 1.16 (0.93-1.44) | 1.00 (Ref) |
| Model 3 | 1.36 (1.07-1.73) | 0.94 (0.74-1.20) | 1.06 (0.84-1.33) | 1.00 (Ref) |
| **Cardiovascular mortality** |  |  |  |  |
| No. of cases | 40 (7%) | 38 (5%) | 33 (3%) | 31 (2%) |
| Model 1 | 1.44 (0.88-2.33) | 1.27 (0.78-2.05) | 1.31 (0.80-2.15) | 1.00 (Ref) |
| Model 2 | 1.38 (0.84-2.27) | 1.37 (0.84-2.23) | 1.29 (0.78-2.13) | 1.00 (Ref) |
| **Cardiovascular events** |  |  |  |  |
| No. of cases | 107 (19%) | 114 (15%) | 108 (8%) | 137 (7%) |
| Model 1 | 1.48 (1.14-1.92) | 1.23 (0.96-1.89) | 1.14 (0.88-1.47) | 1.00 (Ref) |
| Model 2 | 1.49 (1.14-1.94) | 1.22 (0.94-1.58) | 1.17 (0.91-1.52) | 1.00 (Ref) |
| Model 3 | 1.26 (0.94-1.68) | 1.05 (0.80-1.39) | 1.13 (0.87-1.47) | 1.00 (Ref) |

Hazard ratios and 95% confidence intervals derived from Cox proportional hazard models.

Abbreviations: 25(OH)D: 25-hydroxyvitamin D, dp-ucMGP: dephosphorolated uncarboxylated matrix gla protein. To convert 25(OH)D to ng/mL divide by 2.5.

25(OH)D ≥ 50: sufficient vitamin D status, dp-ucMGP ≥361 pmol/L: vitamin K deficiency.

Model 1 adjusted for age, sex, and a cosinor model to account for time of the year

Model 2 includes model 1 plus smoking (never/former/current), body mass index (kg/m^2^), education (3 categories), frequency of sports (3 categories), systolic blood pressure (mm Hg), glucose (mmol/L)

Model 3 includes model 2 and estimated glomerular filtration rate (mL/min/1.73m^2^), blood pressure lowering and cholesterol lowering medication use, prior cardiovascular disease
